# Fluctuations in Daily Happiness and Nervousness Based on Depressive and Anxious Symptoms in Adolescents or Young Adults Across 3 Latin American Cities: Experience Sampling Study

**DOI:** 10.2196/65732

**Published:** 2025-06-19

**Authors:** Ana L Vilela-Estrada, David Villarreal-Zegarra, Nikol Mayo-Puchoc, Nicola Holt, Ángela Flórez-Varela, Catherine Fung, Karen Ariza-Salazar, Fernando Luis Carbonetti, Sumiko Flores, Adriana Carbonel, Natividad Olivar, Carlos Gomez-Restrepo, Luis Ignacio Brusco, Stefan Priebe, Francisco Diez-Canseco

**Affiliations:** 1 CRONICAS Centre of Excellence in Chronic Diseases, Universidad Peruana Cayetano Heredia, Lima, Peru; 2 Department of Biomedical Informatics, School of Medicine, University of Utah, Utah, United States; 3 Instituto Peruano de Orientación Psicológica, Lima, Peru; 4 Department of Health and Social Sciences, University of the West of England (UWE), Bristol, United Kingdom; 5 Department of Clinical Epidemiology and Biostatistics, Pontificia Universidad Javeriana, Bogotá, Colombia; 6 Unit for Social and Community Psychiatry, Wolfson Institute of Population Health, Queen Mary University of London, 58 Turner Street, London, E1 2AB, United Kingdom, 44 7525862392; 7 Department of Psychiatry and Mental Health, School of Medicine, University of Buenos Aires, Buenos Aires, Argentina; 8 Department of Psychiatry and Mental Health, Pontificia Universidad Javeriana, Bogotá, Colombia; 9 Hospital Universitario San Ignacio, Bogotá, Colombia; 10 Unit for Social and Community Psychiatry, East London NHS Foundation Trust, London, United Kingdom

**Keywords:** experience sampling method, mood variability, mood swings, happiness, pediatric, adolescence, teenager, young adult, mental health, mental illness, mental disorder, mental health resources, Latin America, Colombia, Argentina, Peru, mobile phone

## Abstract

**Background:**

Experience sampling methods (ESMs) have been used in clinical research to collect data on emotional and behavioral states in real-life contexts among different populations. Although the use of ESMs in mental health has increased, it has not been applied to larger samples of young people in disadvantaged urban settings.

**Objective:**

This study aimed to determine the extent to which mood status scores (happiness and nervousness) vary during a week, as a function of having or not having symptoms of depression or anxiety, in a sample of adolescents and young adults in the cities of Buenos Aires, Bogotá, and Lima. A secondary objective was to identify factors associated with mood scores, including sociodemographics, quality of life, and daily activities.

**Methods:**

This study was part of the Building Resilience and Resources to Reduce Mental Distress in Young People in Latin America research program, which focuses on mental health resources for young people. Participants (n=143) aged 15‐24 years completed daily ESM assessments over a week using the mobile app, resulting in 5246 reports. Data were analyzed using descriptive analyses with 2-tailed *t* tests and chi-square tests, and multilevel linear regression was used to examine associations between depressive or anxiety symptoms, mood variability, and factors influencing mean mood. Finally, Spearman correlation assessed the relationship between happiness and nervousness.

**Results:**

The analysis revealed that depressive or anxiety symptoms were not significantly associated with increased variability in mood scores (happiness *P*=.40 and nervousness *P*=.84). However, males exhibited greater variability in happiness and nervousness scores (*P*<.001) than females. Additionally, young people showed higher variability in nervousness than adolescents (*P*=.02). Regarding average happiness scores, young adults reported higher average happiness than adolescents (*β*=.604; *P*=.003). Engaging in structured activities (eg, sports, music lessons, and dance classes) was associated with increased happiness (*β*=.266; *P*=.01). In contrast, instrumental activities (eg, cleaning, shopping, meal preparation, or taking medication; *β*=−.144; *P*=.02) and work-related tasks (*β*=−.205; *P*=.01) were linked to lower happiness and higher nervousness (*β*=.387; *P*<.001). Quality of life was positively correlated with happiness (*β*=.486; *P*<.001) and negatively correlated with nervousness (*β*=−.273; *P*=.005). Finally, as for average scores, a strong negative correlation was found between happiness and nervousness (rs=−0.92; *P*<.001). The simple multilevel analysis showed that for each point of happiness, nervousness decreased by 0.45 points (95% CI −0.48 to −0.42; *t*_3_=−41.7; *P*<.001; SE 0.01).

**Conclusions:**

Our study reveals that depressive and anxiety symptoms do not significantly affect the variability in predicted happiness and nervousness scores. However, we observed that demographic factors, such as gender and age, play a role in emotional variability.

## Introduction

### Background

Adolescence and young adulthood are crucial periods of social, behavioral, and psychological development and change [1]. However, these periods are also associated with increased vulnerability to mental health problems, with most functional mental disorders typically manifesting before the age of 25 years [2]. According to the World Health Organization, approximately 14% of young people worldwide experience mental health problems, with anxiety and depression being the most prevalent [3]. These conditions contribute to approximately 13% of the total health burden in this age group [4], and they are more likely to experience depression and anxiety than older adults [5]. As a result, the World Health Organization has identified the reduction of depression and anxiety in adolescents as a key priority, particularly in low- and middle-income countries [6,7]. This includes Latin America, where adolescents and young people living in large cities are more exposed to risk factors for developing anxiety and depression [8,9]. Some studies in Latin America have estimated that the prevalence of depression or anxiety among young people varies, such as 17% in Colombia [10], 16% in Peru [11], and 20% in Argentina [12].

Evidence suggests that researchers and health professionals should join forces to implement integrated actions to understand the behavior of the incidence and prevalence of mental health problems in young people as one of the first steps in the prevention chain [13]. This should complement retrospective data analysis, which is vulnerable to attrition and recall bias [14], with techniques that explore the triggering of symptoms, considering contextual and social factors and elements of everyday life. In this sense, collecting data on people’s experiences and mental states in their daily lives is proposed from psychological and social science research.

The experience sampling method (ESM) is a daily technique of brief assessments that allows data collection in daily life [15]. The ESM uses repeated self-reports through a short questionnaire that participants complete when they receive a signal (eg, phone notifications). The reports assess participants’ experiences and behavior in real time, in their natural environment, and at multiple time points throughout their daily lives [15]. The ESM has several methodological advantages: (1) it improves accuracy and ecological validity, (2) it provides an assessment of participants in their natural environment in real time and at multiple points, and (3) it offers a large and dynamic variation in data analysis and reporting [16-18]. Statistically, ESM studies typically generate substantial data, as they can record multiple measures per day over several days. Consequently, ESM data can be used at a descriptive level (eg, estimating the average of a specific variable) as well as at a more complex level (eg, examining variability in the relationships between variables across different groups) [19].

The ESM has been used in clinical research as a tool for a range of psychological, psychophysiological, cognitive, and behavioral data in real-life contexts in diverse populations [18,20,21], including adolescents and young people [22,23]. Compared with traditional techniques (eg, cross-sectional or longitudinal studies), ESM methodology limits recall bias and captures individual variation in mental health indicators in everyday life [18,24]. Multiple measures of an individual using the ESM help to explore affective variability in more detail and identify the context in which adaptive emotional regulation is used [24].

Mood status variability is the changes and fluctuations in how we feel emotionally over time [25]. High emotional variability can disrupt an individual’s well-being and trigger emotion dysregulation [25]. In this way, studies frequently focus on the role of basic emotions (eg, happiness, nervousness or anxiety, anger, and sadness) because of their relationship to well-being or mental health problems [26]. This variability tends to occur to a greater extent during adolescence [25]. Capturing these variabilities may aid in the development of future assessments of mental health symptoms. Indeed, longitudinal studies have highlighted the importance of mood variability, given that high levels of “negative” emotions (ie, emotional inertia) may contribute to anxiety and depressive symptoms [26,27]. Research has also found that mood variability is associated with differences in support from others; for example, adolescents reported higher levels of negative mood when they perceived less support from their parents [19].

Moreover, applying this methodology to adolescents highlights the association between mental health indicators and the natural context through the average daily scores. Some studies have reported that adolescents with higher levels of negative mood (eg, depressive symptoms, stress, and negative affect) spend more time alone and at home and have reduced physical activity. Other studies have reported that physical activity [20,24,25], engaging in artistic activities, and being outdoors are associated with higher levels of affective well-being [23,28-30].

### Objective

Although the use of the ESM in mental health is becoming more widespread, it has not been applied to larger samples of adolescents and young adults in deprived urban areas of Latin America. Therefore, this study used the ESM to determine the extent to which mood status scores (happiness and nervous) vary during a week, as a function of having or not having symptoms of depression or anxiety, in a sample of adolescents and young adults in the cities of Buenos Aires (Argentina), Bogotá (Colombia), and Lima (Peru). A secondary aim was to identify factors associated with mood scores, such as sociodemographics, wellness factors (quality of life), and daily activities.

## Methods

### Study Design

This study is part of the building resilience and resources to reduce mental distress in young people in Latin America (OLA) research program, designed to identify the personal and social resources that help young people prevent and recover from mental distress [31]. We conducted an ESM study in 3 Latin American countries. This study was designed according to the CREMAS (Adapted Strengthening the Reporting of Observational Studies in Epidemiology Checklist for Reporting Ecological Momentary Assessments Studies) checklist [32], which is an adapted STROBE (Strengthening the Reporting of Observational Studies in Epidemiology) Checklist for Reporting Ecological Momentary Assessments.

### Ethical Considerations

This study’s protocol and all procedures were approved by the Institutional Review Board of Universidad de Buenos Aires (10-02-2020), Pontificia Universidad Javeriana (FM-CIE-1138‐20), and Universidad Peruana Cayetano Heredia (Constancia 581-33-20). Furthermore, it received approval from the Research Ethics Committee of Queen Mary University of London (QMERC2020/02).

In line with ethical guidelines, all young adult participants completed an informed consent form, adolescents completed an informed assent, and their parent or legal guardian provided informed consent. As for the consent procedure, participants were informed about the nature of this study before their participation. They were provided with a detailed explanation of the activity, and those who were willing to participate gave oral or written consent, depending on the specific requirements of this study.

Regarding data privacy and confidentiality, all study data were collected anonymously. No personally identifiable information was linked to the data, and steps were taken to ensure that participants’ confidentiality was maintained throughout the research process.

Compensation for participation varied by country. In Peru, participants were offered a voucher redeemable at a chain shop, valued at 50 soles (at an approximate exchange rate of PEN 1=US $0.27). In Colombia, participants received a voucher worth 140,000 Colombian pesos (COP 1=US $0.00021), while in Argentina, compensation was given in the form of cash worth 2000 Argentine pesos (ARS 1=US $0.0053). The compensation was provided to thank participants for their time and involvement in this study.

### Participants

The sample consisted of participants between adolescents (15 and 16 y) and young adults (20 to 24 y) from a cohort of the OLA research program from Buenos Aires, Bogotá, and Lima [31]. This research program focused on key developmental stages. This study included 2 groups; the first group comprised individuals aged 15‐16 years, as mental health issues often emerge during this period [33]. The second group included young adults aged 20‐24 years, a stage following adolescence but still relevant for the recurrence of mental health disorders [34]. Participants aged 17‐19 years were deliberately excluded to concentrate on the distinct stages of early adolescence and young adulthood, both of which provide valuable insights into mental health outcomes.

The recruitment of OLA targeted areas within the poorest 50% of neighborhoods, as identified using the Human Development Index [35] in Bogotá and Lima, and the Unsatisfied Basic Needs Index in Buenos Aires [36]. This approach aimed to ensure that the sample was representative of disadvantaged populations in these urban areas. Additionally, the recruitment was carried out through collaborations with service care, community organizations, schools, and youth groups in each of the cities.

This study excluded people with severe mental health problems (such as psychosis, bipolar disorder, and schizophrenia), cognitive impairments, illiteracy, or who were users of Huawei (Huawei Technologies Co, Ltd) phones due to issues that arose during the ESM testing phase. Additionally, participants were excluded if they reported a gender other than male or female and if they did not complete the data on the scales used.

Research assistants initially contacted 359 potential participants in the OLA program, primarily via phone calls and messaging. Of these, 7.8% (28/359) were not interested in participating, 10% (36/359) could not be contacted, 19% (68/359) were excluded due to using Huawei phones, 7.5% (27/359) did not complete the informed consent process, and 13.1% (47/359) did not complete training on app usage.

The average time between participation in the OLA cohort baseline questionnaire and enrollment in this study was 107.6 (SD 29.7) days. For further details, please refer to the main publication regarding this activity [30].

### Procedures

Participants who met the inclusion criteria were invited to participate in this study, and informed consent or assent was obtained from those who agreed. Following consent, participants attended a briefing session where the eMoodie (eMoodie Ltd) app was installed on their phones. During this session, they were trained to use the app to complete the Experience Sampling Questionnaire (ESQ).

The eMoodie app, designed specifically for youths, facilitates experience sampling techniques by delivering notifications at random intervals [37]. Over 7 days, participants received notifications to complete ESQs. Notifications for participants aged 15‐16 years were scheduled 5 times daily on school days (excluding school hours) and 8 times daily on weekends. For participants aged 20‐24 years, notifications were sent 8 times daily throughout the 7 days [30]. Their availability was considered to adjust notification times.

Notifications were sent as pop-up alerts on participants’ phones, accompanied by a sound alert. Each notification prompted participants to complete an ESQ, which included questions to assess their current mood. Follow-up was conducted throughout this study to ensure compliance. If participants did not respond to notifications during the first half of the day (morning or afternoon), a follow-up call was made to check their progress. If they had not completed the ESQs on day 2, they were contacted again the next day. Additionally, a follow-up call was made if participants completed less than 50% of the ESQs on days 1 and 2. Participants who had responded to more than 50% of the notifications on day 2 were not contacted. Additionally, support was provided as needed, including app troubleshooting and rescheduling of notifications. No extensions were given to participants who missed notifications, as the study adhered to a strict 7-day timeline.

The final dataset was cleaned to remove invalid responses, such as duplicates or ESQs completed in less than 1 second, indicating “skipping,” and the study protocol specified that only participants who completed at least 40% of the notifications (compliance rate) were included in the final dataset [31]. After data cleaning, the mean compliance rate was 76%, ranging from 34% to 100%.

### Instruments

#### Basal Measurements

As part of the OLA cross-sectional study [38], all participants completed a questionnaire (online or paper) that included the following measures.

The Patient Health Questionnaire-8 (PHQ-8) was used to measure the severity of depressive symptoms. Eight items are on a 4-point Likert scale (0=not at all, 1=several days, 2=more than half of the days, and 3=almost every day). Participants were considered to have depressive symptoms if they had a cutoff score of 10 or more [39].

The General Anxiety Disorder-7 (GAD-7) was used to measure the severity of anxiety symptoms. It has 7 items on a 4-point Likert scale (0=not at all, 1=several days, 2=more than half of the days, and 3=almost every day). Participants were considered to have anxiety symptoms if they had a cutoff score of 10 or more [40].

Our study categorized participants into 2 groups: those with depressive or anxious symptoms and those without symptoms. They were considered to have depressive or anxious symptoms if their scores on the PHQ-8 or GAD-7 were 10 points or more [39,40], respectively.

The Manchester Short Assessment of Quality of Life (MANSA) was used to measure satisfaction with life, work or unemployment, financial situation, number and quality of friendships, sex life, leisure activities, housing, personal safety, people living with others or living alone, family relationships, physical health, and mental health. Satisfaction is measured using a 7-point Likert scale ranging from 1=totally dissatisfied to 7=totally satisfied [41].

As for sociodemographic characteristics, information was collected on participants’ age, gender (male or female), country (Argentina, Colombia, or Peru), main occupation (work, study, housewife, other, or no current occupation), if currently working (no or yes), and education level (primary and high school education, or technical and university education).

#### ESM Component

The following ESQ questionnaire [30] consisted of the following questions:

1. “What are you doing right now?” for a free description of the main activity being performed. Responses were open text in Spanish and were first organized into simple codes for activities such as walking, eating, bathing, etc. The codes were then grouped into 6 broad categories based on categories identified in the literature: (1) Basic activities: activities related to self-maintenance and self-care. Some examples are eating, dressing, personal care and grooming, mobility, etc [42]. (2) Instrumental activities: these are more complex than previous ones and allow people to adapt to their environment. Examples include managing finances, cleaning the home, shopping, preparing meals, taking medication, etc [42]. (3) Relaxation activities: this category refers to more informal and unstructured activities that young people engage in to relax, unwind, or simply pass the time without a specific purpose. This can include activities such as watching television, listening to music, chatting with friends, playing video games, reading for pleasure, taking a nap, etc [43]. (4) Structured activities: these are organized, planned, and have a specific purpose. Generally, these activities are scheduled in advance and have a set timetable, such as sports practice, music lessons, dance lessons, academic tutoring, etc [44]. The latter type of activity included (5) work and (6) educational activities.
2. “How happy or sad do you feel right now?” answered with a 7-point scale from 1=very happy to 7=very sad.
3. “How nervous or calm are you right now?” answered with a 7-point scale from 1=very nervous to 7=very relaxed and calm). In this case, we reverse the scores so that higher scores represent higher nervousness levels.

### Statistical Analyses

#### Characteristics of the Participants

A descriptive analysis was conducted based on the sociodemographic characteristics and variables of interest of the participants. The analysis was performed for the total sample and stratified by the presence or absence of depressive or anxious symptoms. For numerical variables, a 2-tailed Student *t* test was used to assess differences in sociodemographic characteristics based on the presence of depressive or anxious symptoms. Additionally, for categorical variables, a chi-square test was performed to evaluate the association between depressive or anxious symptoms with categorical variables.

#### Average Levels of Happiness and Nervousness

Data were reviewed to identify and remove invalid observations and duplicates. The average compliance rate after cleaning was 76% [30]. A multilevel linear regression analysis was conducted to examine the association between having depressive or anxious symptoms (dichotomized exposition 0 and 1) and the dimensions of happiness (ranging from 1 to 7) and nervousness (ranging from 1 to 7) as criterion variables. We used restricted maximum likelihood. We considered a composite exposure to be the presence of any conditions for the presence of depressive or anxiety symptoms. This analysis considered the nested structure of the data. The first level represented each measurement of happiness and nervousness, averaging 5 to 8 measurements daily. The second level represented each day, with a total of 7 days. Finally, the third level corresponded to each participant, 143 participants (approximately 50 for each country). We consider the presence of depressive or anxious symptoms, gender, age group, quality of life, educational level, and whether the participant is currently working as fixed effects. Additionally, we consider the happiness and nervousness scores as random effects, so we consider these variables using a random intercept and fixed slopes, allowing each participant to have their baseline level of both variables. Further, our analysis controls the time between self-report measures, as we expect measures of happiness and nervousness taken closer together in time to be more correlated than those taken further apart [45].

The use of a multilevel linear regression model accounted for the variability both between measurements taken on each day and between participants, allowing us to capture the hierarchical nature of the data and to examine the association of depressive and anxious symptoms with the dimensions of happiness and nervousness, also adjusting for relevant sociodemographic factors. The effect size was estimated using the adjusted coefficient and a 95% CI. Statistical significance was assessed at a significance level of less than .05. A random effects model was used to account for the nested structure of the data.

#### Variability in Predicted Happiness and Nervousness Scores (Main Analysis)

Our study assessed the variance of the predicted scores for happiness and nervousness using the previous multilevel linear analysis results. We examined whether there were significant differences in the variances of these predicted scores between different groups, such as gender and age groups, to determine whether the variances were equal or differed significantly between them.

Our ANOVA focuses on a robust test for equality of variances. Significant values mean that the variances of the tested groups are different (*P*<.05), and therefore, the scores of 1 group have more variability than another. The Levene test analysis assumes that the scores are independent [45]. Although our data are nested, we considered it appropriate to use this test as it was applied to the predicted values of happiness and nervousness obtained from the linear multilevel model. These predictions were adjusted for covariates, including both the random and fixed effects of the model. This consideration ensures that the predictions adequately reflect variations in the data adjusted for these covariates.

#### Relationship Between Happiness and Nervousness

We conducted a correlation analysis using the predicted scores obtained from the full multilevel model to assess the correlation between these 2 variables. The Kolmogorov-Smirnov normality test with Lilliefors correction identified that the normal distribution assumption was unmet. We performed a correlation analysis between predicted happiness and nervousness scores using Spearman correlation coefficient because a nonnormal distribution was assumed, as these are categorical ordinal values. We used the cutoff of the weak (>0.2), moderate (>0.5), and strong correlations (>0.8).

Additionally, our study conducted a simple multilevel analysis to explore the relationship between happiness and nervousness, assuming the same standards as previously mentioned. This analysis adopts a model incorporating a fixed slope, random intercept, and random effects for happiness and nervousness. We do not use any covariates.

## Results

### Characteristics of the Participants

Initially, 151 individuals participated in the original study; however, 3 participants were excluded for reporting different genders, 4 participants were excluded for not providing MANSA data, and 1 participant was excluded for not reporting education level. Therefore, this study’s sample consisted of 143 participants from Argentina (n=48, 33.6%), Colombia (n=46, 32.2%), and Peru (n=49, 34.3%). The mean age of the participants was 18.6 (SD 3.4) years.

The participants were reported over 7 days, and a total of 5246 reports were collected or completed. Regarding the activities performed by the participants, relaxation activities were the most common (n=1904, 36.3%), followed by basic activities (n=1033, 19.7%). Most participants were women (n=85, 59.4%), exhibited depressive or anxious symptoms (n=86, 60.1%), had studied as their primary occupation (n=105, 73.4%), and were not current smokers (n=104, 72.7%). These activities and the sociodemographic characteristics are shown in Table 1.

**Table 1.** Sociodemographic characteristics and participant activities in adolescents and young adults across 3 Latin American cities (N=143).

			Overall (n=143)	With depressive or anxious symptoms (n=86)	Without depressive or anxious symptoms (n=57)	*P* value
Numerical variable, mean (SD)^a^						
	Age (years)		18.6 (3.4)	18.7 (3.5)	18.5 (3.3)	.75
	MANSA^b^ score		4.65 (1.1)	4.22 (1)	5.26 (0.9)	<.001
	Happiness (weekly average)		4.96 (1.4)	4.78 (1.5)	5.24 (1.3)	<.001
	Nervousness (weekly average)		2.95 (1.6)	3.17 (1.6)	2.64 (1.4)	<.001
Categorical variable, n (%)^c^						
	Gender					.32
		Male	58 (40.6)	32 (37.2)	26 (45.6)	
		Female	85 (59.4)	54 (62.8)	31 (54.4)	
	Country					.98
		Argentina	48 (33.6)	29 (33.7)	19 (33.3)	
		Colombia	46 (32.2)	28 (32.6)	18 (31.6)	
		Peru	49 (34.3)	29 (33.7)	20 (35.1)	
	Age group					.81
		Adolescents (15 and 16 y)	71 (49.7)	42 (48.8)	29 (50.9)	
		Young adults (20 to 24 y)	72 (50.3)	44 (51.2)	28 (49.1)	
	Main occupation					.96
		Work	19 (13.3)	12 (14)	7 (12.3)	
		Study	105 (73.4)	62 (72.1)	43 (75.4)	
		Housewife	7 (4.9)	5 (5.8)	2 (3.5)	
		Other	3 (2.1)	2 (2.3)	1 (1.8)	
		No current occupation	9 (6.3)	5 (5.8)	4 (7)	
	Currently working					.55
		No	104 (72.7)	25 (29.1)	14 (24.6)	
		Yes	39 (27.3)	61 (70.9)	43 (75.4)	
	Education level					.70
		Primary and high school education	98 (68.5)	60 (69.8)	38 (66.7)	
		Technical and university education	45 (31.5)	26 (30.2)	19 (33.3)	
	Activities carried out^d^					
		Basic activities	1033 (19.7)	588 (18.9)	445 (20.8)	.09
		Relaxation activities	1904 (36.3)	1170 (37.6)	734 (34.3)	.01
		Educational activities	557 (10.6)	284 (9.1)	273 (12.8)	<.001
		Structured activities	129 (2.5)	45 (1.5)	84 (3.9)	<.001
		Instrumental activities	884 (16.8)	547 (17.6)	337 (15.8)	.08
		Work activities	309 (5.9)	215 (6.9)	94 (4.4)	<.001

a Student *t* test

b MANSA: Manchester Short Assessment of Quality of Life.

c Chi-square test.

d As the denominator, 5246 evaluations were performed, and only the most relevant activities were reported.

Participants obtained a mean score of 5.05 (SD 1.57) on the happiness scale, which is closer to being happy than to being sad. As for the mean score for nervousness, it was 4.96 (SD 1.42), and this mean score leans more toward nervousness than calmness. Table 1 presents the differences in sociodemographic characteristics between participants with and without depressive or anxious symptoms. Participants with these symptoms showed lower quality of life and happiness scores and higher nervousness scores.

### Variability in Predicted Happiness and Nervousness Scores

According to our multilevel model, having depressive or anxiety symptoms did not result in significant differences in the variability of predicted happiness (*P*=.40) and nervousness (*P*=.84) scores compared to not having symptoms. This means that participants with depressive or anxious symptoms do not show greater variability in their scores between the different measurements; that is, there are no large changes from high to low scores from one measurement to another compared to people without such symptoms.

However, we did observe a relationship between the variations in the predicted nervousness scores and some demographic variables, including gender and age group. Specifically, males and individuals aged 20 to 24 years exhibited higher variability in their nervousness scores than females and adolescents, respectively.

Similarly, differences in the variations of the predicted happiness scores were associated with gender. Thus, male participants exhibited the highest variability in their happiness scores (Table 2).

**Table 2.** Variability in happiness and nervousness scores in adolescents and young adults across 3 Latin American cities. Our analysis used a robust test for equality of variances. Analysis was performed on 143 participants with a total of 5246 experience sampling method reports.

		Happiness			Nervousness		
		SD	W_50_	*P* value	SD	W_50_	*P* value
Gender			107.3	<.001		26	<.001
	Female	0.474			0.373		
	Male	0.561			0.408		
Age group			0.4	.51		5.2	.02
	Adolescents (15 and 16 y)	0.524			0.397		
	Young adults (20 to 24 y)	0.539			0.420		
Depressive or anxious symptoms			0.7	.40		0	.84
	No	0.492			0.354		
	Yes	0.505			0.342		
Day			0.6	.70		0.5	.77
	Monday	0.539			0.418		
	Tuesday	0.536			0.418		
	Wednesday	0.542			0.418		
	Thursday	0.525			0.402		
	Friday	0.527			0.409		
	Saturday	0.527			0.401		
	Sunday	0.555			0.421		

### Average Mean Scores of Happiness and Nervousness

Young adults had higher mean scores of happiness than adolescents (*β*=.604; *P*=.003; 95% CI 0.214 to 0.994). In addition, quality of life scores were associated with higher happiness scores (*β*=.486; *P*<.001; 95% CI 0.334 to 0.637). Regarding specific activities they engaged in during the assessment, participants who were engaged in structured activities had higher mean happiness scores than those who did not engage in these activities (*β*=.266; *P*=.01; 95% CI 0.055 to 0.476). On the contrary, participants who were engaged in instrumental activities (*β*=−.144; *P*=.02; 95% CI −0.263 to −0.026) or work activities (*β*=−.205; *P*=.01; 95% CI −0.362 to −0.048) had lower mean happiness scores than those who were not engaged in these activities (Table 3).

The mean scores of nervousness were negatively associated with quality of life scores (*β*=−.273; *P*=.005; 95% CI −0.463 to −0.084) and were higher among those engaged in work activities (*β*=.387; *P*<.001; 95% CI 0.214 to 0.561) compared to those who did not (Table 4).

**Table 3.** Average happiness levels in adolescents and young adults across 3 Latin American cities. Analysis was performed on 143 participants with a total of 5246 experience sampling method reports.

		Mean predicted		Estimate	SE	*t* test (*df*)	*P* value	Lower^a^	Upper^b^
		No	Yes						
Depressive or anxious symptoms		5.19	4.8	0.103	0.16	0.64 (136)	.52	−0.216	0.421
Gender		5.07^c^	4.88^d^	−0.021	0.14	−0.15 (136)	.88	−0.302	0.26
Age group		4.89^e^	5^f^	0.604	0.2	3.06 (136)	.003	0.214	0.994
Activities									
	Basic activities	4.89	5.22	0.103	0.06	1.77 (4254)	.33	−0.641	0.848
	Relaxation activities	4.97	4.94	−0.072	0.05	−1.31 (4254)	.42	−0.767	0.624
	Educational activities	5	4.58	−0.368	0.07	−5.23 (4254)	.12	−1.262	0.527
	Structured activities	4.95	5.37	0.266	0.11	2.47 (4254)	.01	0.055	0.476
	Instrumental activities	4.97	4.88	−0.144	0.06	−2.39 (4254)	.02	−0.263	−0.026
	Work activities	4.97	4.82	−0.205	0.08	−2.56 (4254)	.01	−0.362	−0.048
MANSA^g^ score (numeric)		—^h^	—	0.486	0.08	6.34 (136)	<.001	0.334	0.637

a Lower: lower bound 95% interval.

b Upper: upper bound 95% interval.

c Male.

d Female.

e Adolescents (15 and 16 y).

f Young adults (20 to 24 y).

g MANSA: Manchester Short Assessment of Quality of Life.

h Not applicable.

**Table 4.** Average nervousness levels (reverse coded) in adolescents and young adults across 3 Latin American cities. Analysis was performed on 143 participants with a total of 5246 experience sampling method reports.

		Mean predicted		Estimate	SE	*t* test (*df*)	*P* value	Lower^a^	Upper^b^
		No	Yes						
Depressive or anxious symptoms		2.7	3.15	0.173	0.2	0.86 (136)	.39	−0.227	0.573
Gender		2.82^c^	3.07^d^	0.144	0.18	0.81 (136)	.42	−0.208	0.496
Age group		5^e^	5.06^f^	−0.431	0.25	−1.74 (136)	.08	−0.921	0.059
Activities									
	Basic activities	3.02	2.73	−0.092	0.07	−1.4 (4254)	.39	−0.923	0.739
	Relaxation activities	3	2.91	0.009	0.06	0.15 (4254)	.91	−0.763	0.781
	Educational activities	2.9	3.49	0.554	0.08	7.11 (4254)	.09	−0.436	1.545
	Structured activities	2.97	2.59	−0.182	0.12	−1.53 (4254)	.13	−0.415	0.051
	Instrumental activities	2.95	3.01	0.117	0.07	1.75 (4254)	.08	−0.014	0.248
	Work activities	2.94	3.29	0.387	0.09	4.38 (4254)	<.001	0.214	0.561
MANSA^g^ score (numeric)		—^h^	—	−0.273	0.1	−2.85 (136)	.005	−0.463	−0.084

a Lower: lower bound 95% interval.

b Upper: upper bound 95% interval.

c Male.

d Female.

e Adolescents (15 and 16 y).

f Young adults (20 to 24 y).

g MANSA: Manchester Short Assessment of Quality of Life.

h Not applicable.

### Relationship Between Happiness and Nervousness

The full multilevel analysis, which includes various covariates, shows a strong and negative correlation between the predicted values of happiness and nervousness (rs=−0.92; *P*<.001; n=5246). Additionally, the simple multilevel analysis found that for each point of happiness, the nervousness score decreased by an average of 0.45 points (95% CI −0.48 to −0.42; *t*_3_=−41.7; *P*<.001; SE 0.01).

## Discussion

### Main Findings and Comparison With Previous Work

This study used the ESM to examine daily mood status variability (happiness and nervousness) in a sample of adolescents and young adults with (n=86/143, 60%), and without (n= 57/143, 40%) anxiety or depressive symptoms in Buenos Aires, Bogotá, and Lima. Our findings revealed that having depressive or anxiety symptoms is not significantly associated with differences in the variability of daily happiness and nervousness scores.

Although some studies have found that mood variability in patients with mental health problems [46-48], mainly in adults with major depressive disorder, was higher than in individuals without mental health problems, there is still much debate about the nature of mood fluctuations in this population. Some studies are consistent with our findings, reporting “emotional inertia,” “emotional inflexibility,” or lower mood variability over time in these individuals. For example, Lamers et al [49] and Nelson et al [50] found no significant differences in mood variability between those with and without mental health issues. Kuppens et al [27] argue that depressive episodes involve high emotional inertia, implying less mood variability, while Shin et al [51] identified that negative experiences correlated with anxiety and depression but did not significantly impact variability.

These mixed findings highlight the complexity of mood variability to mental health, indicating that other factors may also influence these dynamics. Considering this, Pemberton and Tyszkiewicz [52] suggested that the apparent discrepancy between stability and variability in assessments of mood in individuals with mental health problems may be due to the use of different time frames in other studies. It is, therefore, plausible that individuals manifest both short-term mood stability and variability when examined over a more extended period. The mood swings observed in our study may reflect the “short-term stability” of mood in individuals with mental health problems, and it is possible that over a longer period, fluctuations indicating severity or recovery from emotional distress may become more evident. For example, a study of 482 Dutch adolescents followed up 3 times a year for 5 years to observe how their mood varied and how this related to depressive symptoms, delinquent acts, and alcohol consumption. Most of the adolescents showed emotional stability; however, a small group experienced an increase in mood variability during middle adolescence, showing more persistent depressive symptoms and delinquent behaviors [53]. Similarly, 1 study assessed the daily fluctuation in mood (ie, happiness, anger, sadness, and anxiety) with 54 adolescents (9‐12 y) during 4 months at a daily reporting interval. Results evidenced “relatively stable” levels of mood during the first months of the COVID-19 pandemic [54]. Another study of 58 early adolescents from the Netherlands was conducted over 7 days to explore associations between social context and adolescents’ daily mood state. Participants were enrolled in 5 to 8 daily prompts, using the Ecological Momentary Assessment. Early adolescents showed mood variability when they are alone than in peripheral company [55].

However, our results regarding emotional variability reveal a notable divergence from existing literature on gender differences in adolescents and young adults [25,56]. Specifically, we found that males exhibited greater mood variability in happiness and nervousness than females. This variability was particularly pronounced in happiness scores compared to females. This observation contrasts with the common finding in the literature that females typically report greater emotional variability. However, it aligns with studies suggesting that males might experience more intense emotional responses to stimuli that induce happiness, excitement, or perceived challenges [57,58].

Finally, concerning emotional variability, young adult participants exhibited greater variability than adolescents in nervousness scores. Previous studies have shown that greater emotional instability is common during late adolescence and young adulthood, resulting in wider and more rapid mood swings. However, these are not always largely related to psychological or social maladjustment within each adolescent [59,60]. These fluctuations in mood are usually the result of internal or external processes to which a person is more or less sensitive, but they will also involve individual and contextual aspects such as the affective base (the initial emotional state) and the strength of the attractor (the tendency to return to a specific emotional state) [27].

It was identified that there is a significant and negative relationship between the average scores of happiness and nervousness obtained during the follow-up week, meaning that as happiness increases, nervousness tends to decrease, and vice versa [61]. This finding aligns with the theoretical understanding that these two emotions are inversely related and suggests that intervening in one emotion could impact the other [61]. Recognizing this interplay can inform the development of more effective strategies for emotional well-being, potentially incorporating approaches that address both positive and negative emotions to achieve a more balanced emotional state.

We also found that young adults and those with higher perceived quality of life had higher average happiness scores during the follow-up week. Some studies suggest a reduction in subjective well-being during adolescence, which could be attributed to factors such as social pressure, uncertainty about the future, and other typical changes in this life stage [62-64]. As individuals transition into adulthood, they often develop new identities and relationship patterns, potentially leading to increased cohesion and commitment to personal and social goals [65]. Additionally, previous research indicates that higher subjective well-being is associated with more favorable perceptions of various life aspects, such as health, interpersonal relationships, and the work environment [66]. This supports our findings, suggesting that lower quality of life scores are related to higher nervousness levels.

For daily activities reported in the ESM questionnaire by participants during the follow-up week, engaging in structured activities (such as playing sports, doing art activities, having group activities with others, etc) was positively related to happiness scores. This is consistent with previous literature using the ESM, where adolescents reported feeling happier during structured leisure activities due to the opportunity to develop activities of interest and the positive impact on mood improvement in everyday life, and found that time spent with peers particularly reduces symptoms of anxiety and sadness [67,68].

Finally, doing instrumental activities (eg, managing finances, cleaning the home, shopping, preparing meals, etc) was negatively related to happiness, and performing work activities was related to lower happiness and higher nervousness levels. These results could be explained by the fact that instrumental activities may be perceived as a burden or an obligation rather than as rewarding or satisfying [68-71]. Similarly, work activities can be a source of stress and worry for many people. This may be due to work pressure, job demands, or lack of job satisfaction.

### Strengths and Limitations

Using the ESM, this study collects real-time data on emotional and behavioral states in real contexts from young people in urban areas in middle-income countries. The use of the ESM to explore variability in mood was fruitful, as it allowed us to analyze temporal and contextual patterns that, although associative, suggest that engagement in activities (eg, work and instrumental) is important for reducing distress in young people’s daily lives. Additionally, our sample size was relatively higher compared to other similar studies [54,55], enabling us to understand the variability in mood status, however, generalizability should be treated with caution.

However, it is important to consider some study limitations when interpreting the results. A limitation of this study is that data collection occurred an average of 107.6 (SD 29.7) days after participants completed the baseline OLA cohort questionnaire. This time lag may have affected the accuracy of characterizing participants, particularly in differentiating between those with and without depressive or anxiety symptoms, as the data may not fully reflect their emotional states before the start of the week of data collection. Although emotional state was assessed using 2 self-report questions with Likert-type scales to capture different mood extremes (eg, sad-happy or nervous-relaxed), this methodology, although friendly due to the frequency of daily notifications, could have led to participants responding in a way that projected a positive self-image or was aligned with perceived expectations. Furthermore, although the activities performed were assessed, the quality of these experiences (positive or negative) was not considered, nor were other factors that might interact with the activities to influence mood, such as competence or self-esteem.

Finally, although compliance rates were satisfactory, problems with receiving notifications may have affected the participation of some individuals due to issues such as poor or no internet connection during the eMoodie app download, specific technical problems with eMoodie on certain smartphone models, or battery-related problems.

### Implications in Public Health

The findings from this study underscore the importance of developing customized approaches for enhancing emotional well-being, considering the interplay between demographic factors, quality of life, and emotional variability. Further research is needed to investigate how elements such as gender, age, and the nature of daily activities affect emotional experiences. For clinical practice, these results suggest that interventions may be more successful if they promote engagement in structured activities and focus on improving overall quality of life. Understanding variations in emotional responses across different demographic groups can inform the creation of more targeted and individualized strategies. While it is well known that adolescents experience variability in mood state, professionals need to acknowledge that mood variability should not relate to psychological or social maladjustment, given that mood swings seem to be an inherent aspect of adolescent lifestyle [60]. From a policy standpoint, ensuring fair access to opportunities and activities that support emotional health is essential. Policies facilitating access to structured and fulfilling activities could enhance emotional well-being and help mitigate differences in emotional experiences among various groups.

### Conclusions

Our study reveals that depressive and anxiety symptoms do not significantly affect the variability in predicted happiness and nervousness scores. However, we observed that demographic factors, such as gender and age, play a role in emotional variability, with males and individuals aged 20 to 24 years exhibiting greater nervousness fluctuations and males showing higher variability in happiness. Additionally, young adults reported higher average happiness scores than adolescents, and higher quality of life was associated with greater happiness. Structured activities were linked to increased happiness, whereas instrumental and work-related activities were associated with lower happiness and higher nervousness. These findings highlighted the importance of considering demographic factors and the nature of daily activities when evaluating emotional well-being and suggested that enhancing quality of life and engaging in structured activities could contribute positively to emotional health.

## Abbreviations

CREMAS: Adapted Strengthening the Reporting of Observational Studies in Epidemiology Checklist for Reporting Ecological Momentary Assessments Studies
ESM: experience sampling method
ESQ: Experience Sampling Questionnaire
GAD-7: General Anxiety Disorder-7
MANSA: Manchester Short Assessment of Quality of Life
OLA: building resilience and resources to reduce mental distress in young people in Latin America
PHQ-8: Patient Health Questionnaire-8
STROBE: Strengthening the Reporting of Observational Studies in Epidemiology
